# Crash Performance of Strength Gradient Tube Induced by Selective Laser Patterning

**DOI:** 10.3390/ma15196580

**Published:** 2022-09-22

**Authors:** HyungGyu Kim, NamHoai Trinh, JunBeom Kwon, SukJoon Hong, SungHyuk Park, JongHun Yoon

**Affiliations:** 1 Department of Mechanical Design Engineering, Hanyang University, 222, Wangsimni-ro, Seongdonggu, Seoul 04763, Korea; 2 Department of Mechanical Engineering, BK21 FOUR ERICA-ACE Center, Hanyang University, 55, Hanyangdaehak-ro, Ansan 15588, Gyeonggi-do, Korea; 3 Materials Analysis & Evaluation Department, Korea Institute of Materials Science (KIMS), 797, Changwon-daero, Changwon 51508, Gyeongsangnam-do, Korea; 4 School of Materials Science and Engineering, Kyungpook National University, 80, Daehakro, Daegu 41566, Korea; 5 AIDICOME Inc., 55, Hanyangdaehak-ro, Ansan 15588, Gyeonggi-do, Korea

**Keywords:** local hardening, laser, strain rate sensitivity, FEM analysis, auxetic, helical, laser patterning

## Abstract

This paper presents an investigation of the performance of a 22 MnB5 tube after local heat treatment according to a patterning shape under dynamic crash test conditions to propose the patterning shape with the best energy absorption efficiency. Numerical simulations support experimental results to validate the deformation mode during dynamic crash test as well as the strain distribution of the specimen. The helical patterning not only demonstrates the highest axial loading force and energy absorbance in both static and dynamic crash tests, but also can be easily fabricated in a short time. The helical pattern can optimize different pitch sizes according to the thickness and diameter of the cylindrical tube, and it has the highest energy absorption rate with 83.0% in dynamic conditions.

## 1. Introduction

In automotive engineering, energy absorption is a vital consideration for passive security while keeping the vehicle frame lightweight, a need which increases the demand for high-and ultra-high-strength steel. There are three common methods of realizing lightweight automobiles. The first, whereby the body frame and structures are improved using a body-in-white design or identifying alternative materials such as carbon-fiber-reinforced polymers to reduce the vehicle mass. The second adopts an advanced manufacturing technique to improve the strength of the material, such as in thickness-gradient high-strength steel (HSS). In the third, metal-based compound plates are fabricated using continuous pressing and hot press forming (HPF) [1,2]. After conventional HPF, the HSS finally has a martensite microstructure with a total strength of approximately 1.5 GPa. It exhibits low formability and weldability, which presents a significant challenge in joining different parts of dissimilar materials not only in the automotive industry, but also in other industries. While high-strength parts are required to improve automobile crash safety under reduction or without an increase in car weight [3], the crumple zone must maintain a relatively low strength for absorbing collision energy through impact deformation. Hence, hot-stamped parts are mainly used as reinforcement members and passenger zones to maximize the crashworthiness and ensure human safety, such as B-pillars [4], and tailoring panels are used for making crumple zones such as bumper beams.

In general, it is crucial to study flexible and innovative manufacturing technologies that can meet various requirements. Some common methods that were studied and applied, such as tailor heat-treated blank [5], can be categorized as tailored weld blank and tailored rolled blank [6], local hardening or softening [7]. Although tailored HPF has some major advantages, such as good dimensional accuracy, it still has some drawbacks: high investment costs of the press, stamping mold, cooling device, and other auxiliary equipment and low energy efficiency of the furnace [2]. In contrast, the local hardening or softening is more flexible, as it only induces strength in the necessary area by controlling the quenching region. Several local hardening or softening methods have been proposed, including local heating [8], tailor die quenching [9], post tempering [10], and annealing [11]. Vogt et al. [12] proposed local laser softening for panel joining areas via laser treatment before performing welding for press-hardened steel to overcome the spot-welding failure, characterized the influence of the parameters and investigated the mechanical properties of the anisotropic material. Asadi et al. [13] applied a local laser strengthening method in hot-rolled dual-phase steel, which was globally and homogenously deformed with different degrees of pre-strains by cold rolling to correlate the local microstructure and local mechanical properties at different heating temperatures and pre-strains level. Nakagawa et al. [14] presented three tailored tempering processes, including multiple stages without die heating, to manufacture tailored parts: these have both high strength and high ductility zones in an acceptable cycle time. It is promising for actual manufacturing applications. Kim et al. [15] investigated local strengthening using induction heating and water spray quenching in the target region in a B-pillar panel to evaluate the effect of process parameters such as quenching rate, processing time, and heating width, while Li Bao et al. [16] used a partition controllable induction heating-stamping process to obtain high-strength boron-alloyed steel plates in order to realize different microstructures in the test B-pillar part after applying various temperature distribution region patterns. This provided a new method of realizing lightweight automobiles and significantly increasing human safety. Coupard et al. [17] focused on investigating the distribution of axial and circumferential residual stress after induction of quenching plain cylindrical specimens via an X-ray diffraction technique and multi-physics finite element modelling of the entire induction treatment process, which included electromagnetic, thermal, metallurgical, and mechanical phenomena.

Crashworthiness is the parameter used to predict the capability of the structure to prevent massive damage and protect the passenger, and the energy-absorbing component serves as the main structure that can dissipate the most energy during a crash event. Because significant energy can be absorbed by plastic deformation during the progressive fold formation process, an axial crashing test has many important engineering safety applications in terms of the crashworthiness and blast-resistant design of structures.

DiPaolo et al. [18] studied the crash characteristic response of a thin-wall steel box component under quasi-static conditions with different types of AISI steel, while Abramowicz conducted dynamic tests on two different cross-sections of thin-walled square tubes in [19] and circular cylindrical shells in [20] with various lengths to obtain a theoretical prediction and reasonable agreement with the corresponding experimental results. Alomarah et al. [21] performed experiments and simulations to investigate the effects of the crash velocity and the in-plane mechanical properties of auxetic structures subjected to dynamic compression. Ruan et al. [22] examined the dynamic behavior of hexagonal aluminum honeycombs using a finite element simulation with ABAQUS to determine the influences of the honeycomb cell wall thickness and the impact velocity on the mode of localized deformation and the plateau stress. Gunaydin et al. [23] not only investigated the experimental and numerical crashworthiness performance of thin-walled tubes filled with two different auxetic lattice structures having different deformation mechanisms under quasi-static loading, but also made an optimal design for the improvement of the collision performance. Tan et al. [24] compared the crash performance of crash boxes to which auxetic and honeycomb structures are applied with traditional crash boxes and proposed an optimal structure that is filled with auxetic hierarchical cores and covered with square thin-walled tubes. Thus, recently, an auxetic structure having a negative Poisson’s ratio has been widely applied to improve collision performance, but there are few studies on inducing the strength gradient of a material in an auxetic structure using laser patterning.

The conventional hot stamping system is a method of strengthening the entire panel, and since a strength gradient cannot be given to one panel, local hardening methods have been introduced. This includes tailored heating, tailored quenching, and induction heating methods to induce strength variation. In this paper, we introduced helical patterns with local hardening using laser patterning techniques on the cylindrical tube specimen to solve the issue of specimen fabrication time, which is the biggest drawback of laser patterning. We suggested an optimal pitch size to verify the findings are supported by experiments and numerical simulation to secure novelty. Following previous research, the effect of laser patterning on the deformation mode and energy absorption in axial crash tests with 22 MnB5 cylindrical tubes having different types of patterns under static loading conditions was confirmed [3]. This paper is focused on discussing the effects of the laser pattern on the deformation mode and energy absorption of 22 MnB5 cylindrical tubes with various patterning shapes in dynamic crash conditions. Subsequently, an optimal laser pattern was proposed based on the experimental results and numerical simulations. The patterns applied to the as-received tubes were 0°, 90°, grid, honeycomb, auxetic, and helical. By controlling the pattern, the crash performance of the tubes can be tailored and enhanced owing to the martensite transformation with an ultimate tensile strength greater than 1.2 GPa under the heat of the laser treatment.

## 2. Experimental Procedure

**Figure 1:**
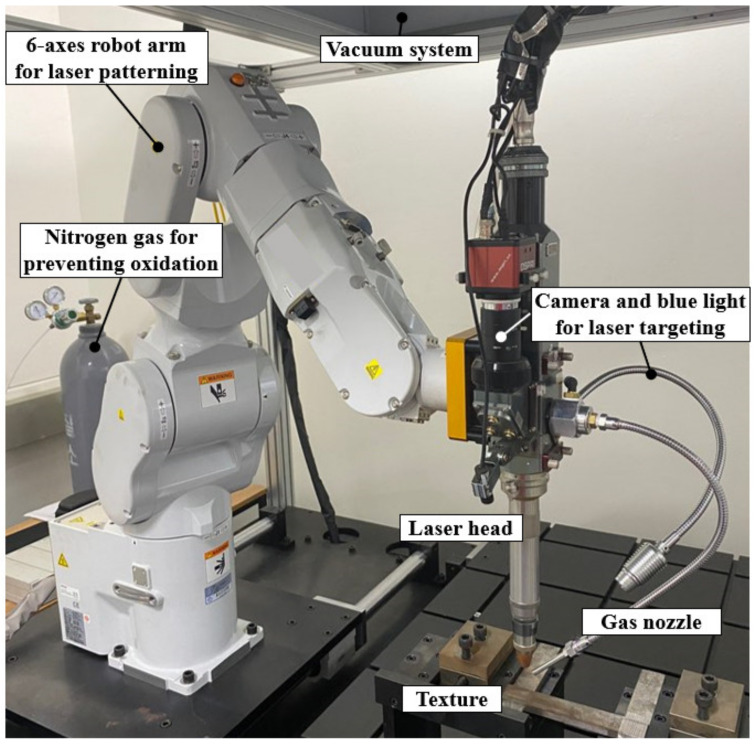
Experimental set-up for the laser patterning.

**Figure 2:**
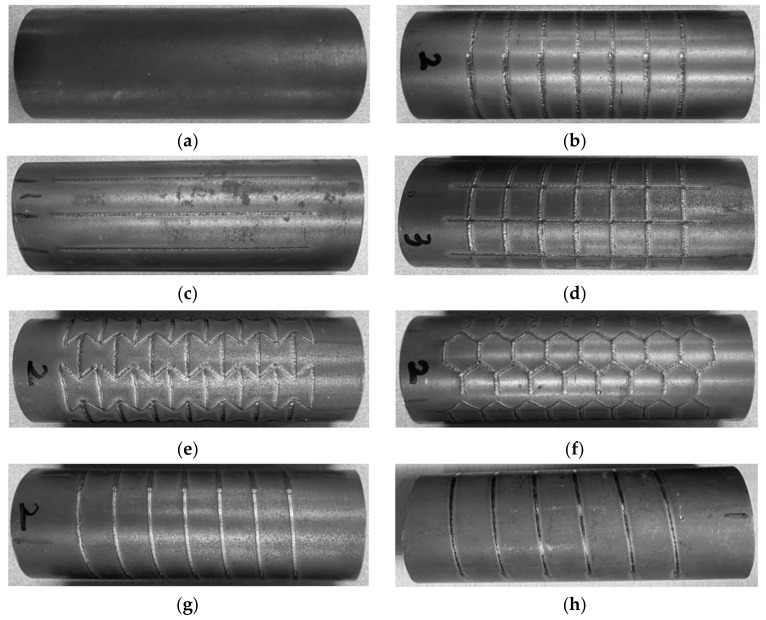
Laser-irradiated specimens with patterning of: (**a**) initial; (**b**) 0°; (**c**) 90°; (**d**) grid; (**e**) auxetic; (**f**) honeycomb; (**g**) helical with pitch of 10 mm; (**h**) helical with pitch of 12.5 mm.

To fabricate the tube with local laser heat treatment, a 22 MnB5 cylindrical steel tube was prepared based on our previous research [3]. This material was selected because it easily undergoes martensitic transformations using an irradiating laser source on a target zone. Figure 1 presents an experimental setup for the laser patterning in which a fiber laser source with a power of 400 W and feed rate of 2.5 mm/s was manipulated using a six -axis robot arm to irradiate specific geometrical patterns such as 0°, 90°, grid, honeycomb, auxetic, and helical patterns. These irradiated patterns were categorized into two groups: conventional patterns including 0°, 90°, grid, and helical patterns; complex patterns including auxetic and honeycomb formations, as shown in Figure 2. Although the fabrication of a complex pattern required much more time, it exhibited superior absorbed energy in the quasi-static test, thus enhancing the material strength of the tube [3,25]. The honeycomb structure can be found in many natural materials such as wood, bone, and beehives and has drawn much attention for many years. The auxetic pattern, is also special and has drawn the attention of many researchers because of its distinctive Poisson’s ratio. Poisson’s ratio is defined as the ratio between the lateral and axial strains in the tensile test, and it is an important mechanical property, especially in the fields of structural stability and microstructural transitions at the microscopic level [26]. The majority of conventional materials have a positive Poisson’s ratio, which indicates that they are subjected to shrinkage in the lateral direction and elongation along an axial direction under a tensile stress. In contrast to conventional materials that have a positive Poisson’s ratio, auxetic materials exhibit a negative Poisson’s ratio, which enhances their shear stiffness, impact resistance, indentation hardness, fracture toughness, and resilience [25]. Because of these advantages, the honeycomb and auxetic structures were selected as promising candidate patterns for the comparison with other conventional patterns for improving the tube crashworthiness.

**Figure 3:**
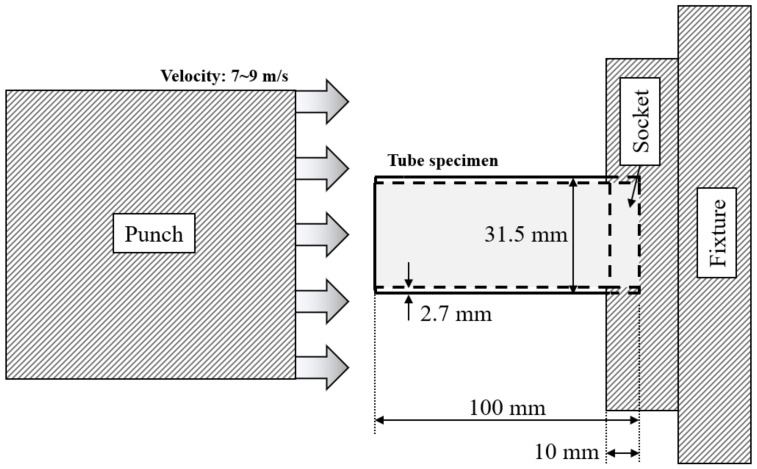
Schematic of dynamic crash test.

**Figure 4:**
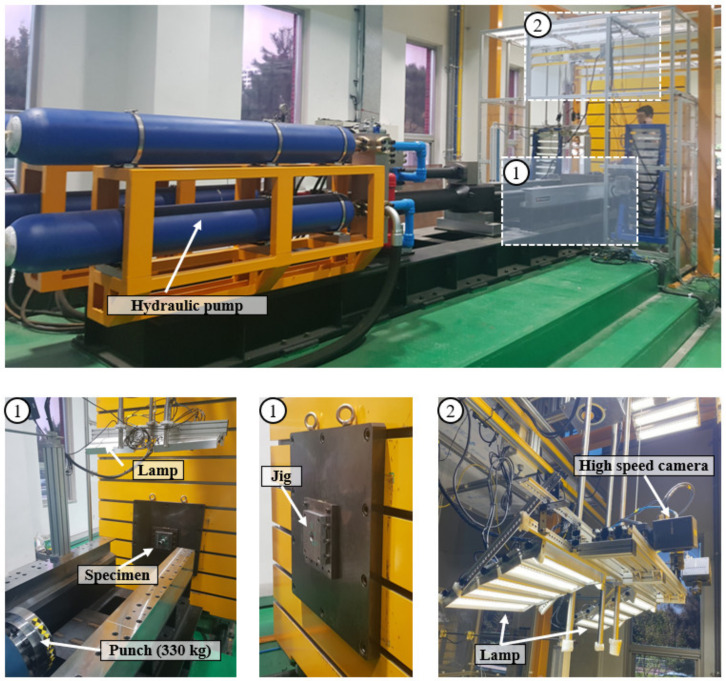
Experimental set-up for dynamic crash test.

Figure 3 presents a schematic of an axial crash test under dynamic conditions, with a 7–9 m/s velocity for the experimental validation of the patterned tubes. The as-received tube had a length of 100 mm, outer diameter of 31.5 mm, and thickness of 2.7 mm, and it was inserted into the socket at a depth of 10 mm. Figure 4 shows an experimental setup of a dynamic crash test using a hydraulic cylinder, consisting of a laser sensor for measuring the speed of the punch, a lamp, and a high-speed camera (Photron’s UX50 model) with a maximum 5000 frame rate and 1280 × 1024 pixels for measuring the deformation mode of the specimen; a die set for fixing the specimen; and a 330-kg punch for simulating the crash of a cylindrical tube specimen. A hydraulic crash tester was required to transmit a high kinetic energy; however, as the temperature of the oil in the cylinder increased owing to the repeated use of the hydraulic cylinder, speed control became difficult, and thus, the range of the speed was inevitably provided [27].

## 3. Effects of Laser Patterning

### 3.1. Deformation Behaviour

An axial crash test was performed to investigate the absorbed energy and deformation behavior with respect to the punch stroke [3]. Before discussing the deformation behavior, we need to know the terminology used in previous research [3,28]. 

**Figure 5:**
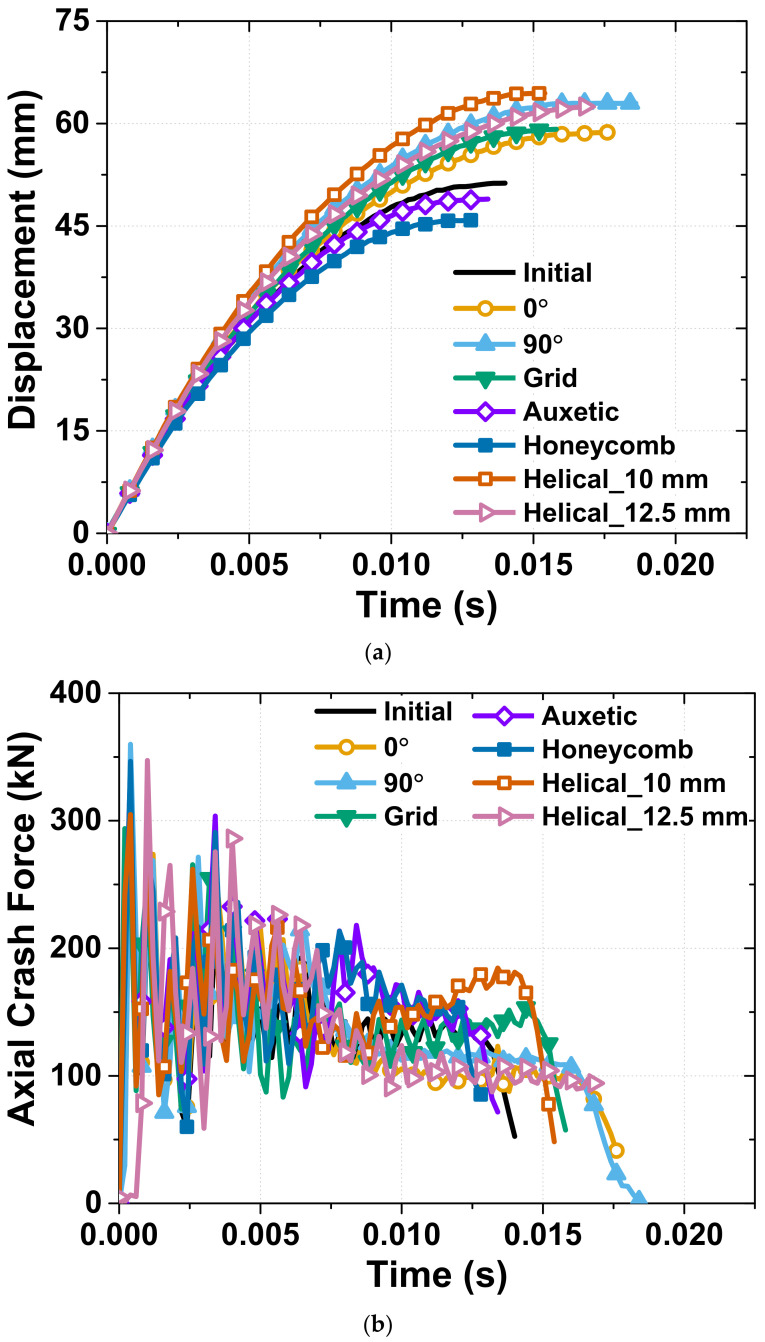
Experimental results of dynamic crash test: (**a**) displacement; (**b**) axial crash force according to time.

The load–displacement data was used for measuring the axial crash force according to the tube shortening in real time during the crash test, as illustrated in Figure 5. Figure 5 describes the displacement and axial crash force with respect to time in the dynamic crash test. The load–displacement data were calculated using the following procedure. Step 1 comprised recording the shortening of the tube with time by measuring the displacement of the hydraulic punch during the crash test. In Step 2, we collected the axial crash force data that is measured using the crash test equipment; finally, we combined the two data points of Steps 1 and 2 to obtain the load–displacement result. 

**Figure 6:**
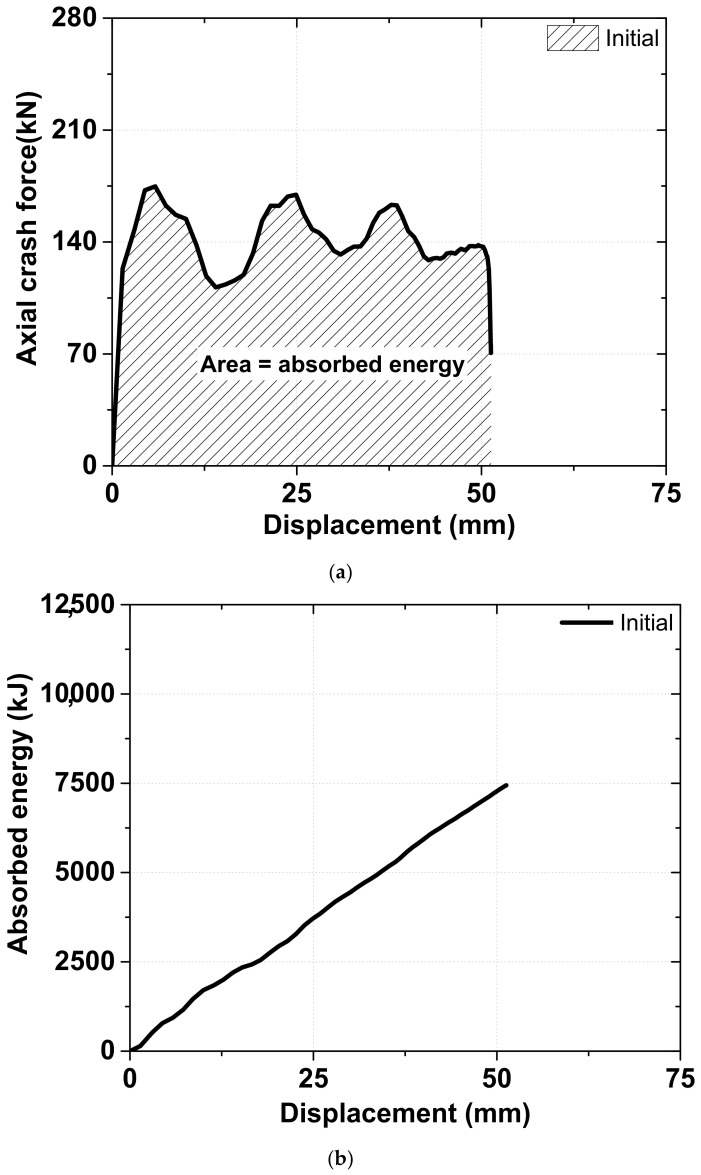
An example of the absorbed energy calculation method using the initial specimen: (**a**) integration area for calculating absorbed energy; (**b**) absorbed energy with respect to displacement.

The second variable determined was the absorbed energy, which is equal to the area under the load–displacement curve and can be calculated using numerical integration [28], as illustrated in Figure 6. Figure 6 shows how to calculate the absorbed energy in the axial crash force according to the displacement, using initial specimen. The third is the deformation mode, which is generally categorized into the concertina, diamond, and complex modes. While the concertina mode indicates uniform folding along the radial direction, the diamond mode exhibits axisymmetric deformation with one side of the fold directed inward and the other directed outward [3].

**Figure 7:**
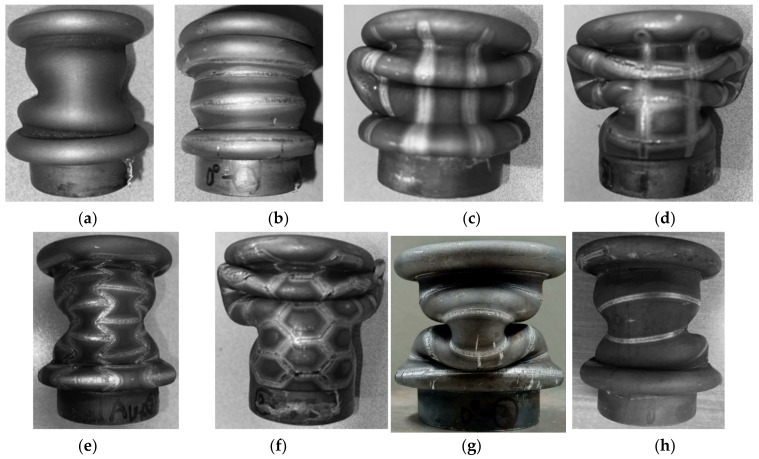
Deformed shapes with respect to laser patterning: (**a**) initial; (**b**) 0°; (**c**) 90°; (**d**) grid; (**e**) auxetic; (**f**) honeycomb; (**g**) helical with pitch of 10 mm; (**h**) helical with pitch of 12.5 mm.

After the dynamic punch stroke of 40 mm with speed varied from 7–9 m/s, the deformation mode of each specimen is showed in Figure 7 and summarized in Table 1. The concertina mode and diamond mode are denoted as C and D respectively.

**Figure 8:**
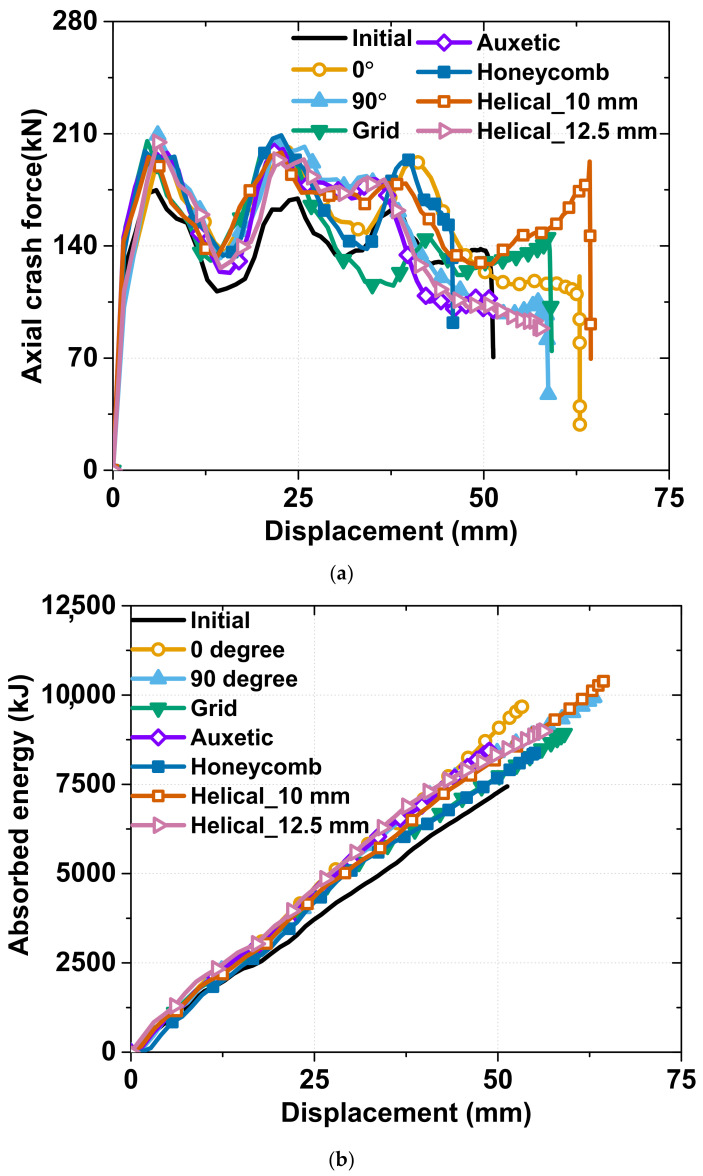
Performance comparison in dynamic crash tests according to laser patterning: (**a**) load–displacement curve; (**b**) total absorbed energy.

Because of the dynamic crash effect, the effect of the patterning was weakened, which is expressed by the axial crash force–displacement graph shown in Figure 8a. In this graph, the initial first and second fold in the majority of the specimens occurred in the concertina mode, and the crash force–displacement graph thus tended to be similar; the axial crash force that can be withstood by the specimen improving by up to 50% compared to that of the as-received tube. At the third folding, the effect of the patterning shape was clear, and the specimens could be divided into two groups. The specimens of group 1 had the highest value and included the 0°, honeycomb, and helical specimens. Group 2 included the 90°, optimal, and auxetic patterning specimens, whose graphs were similar in both trend and value. The grid patterning specimen exhibited a different tendency from the others.

In the absorbed energy chart in Figure 8b, the helical patterning specimen achieved the highest value because it not only had the highest axial crash force but also the greatest delay with the largest displacement. Although the absorbed energy values of the 0° and 90° patterning specimens were not as high as those of the helical specimen, they showed considerably high values among the rest of the others, and the auxetic, honeycomb, and grid patterning specimens exhibited similar values.

### 3.2. Strain Rate Sensitivity

**Figure 9:**
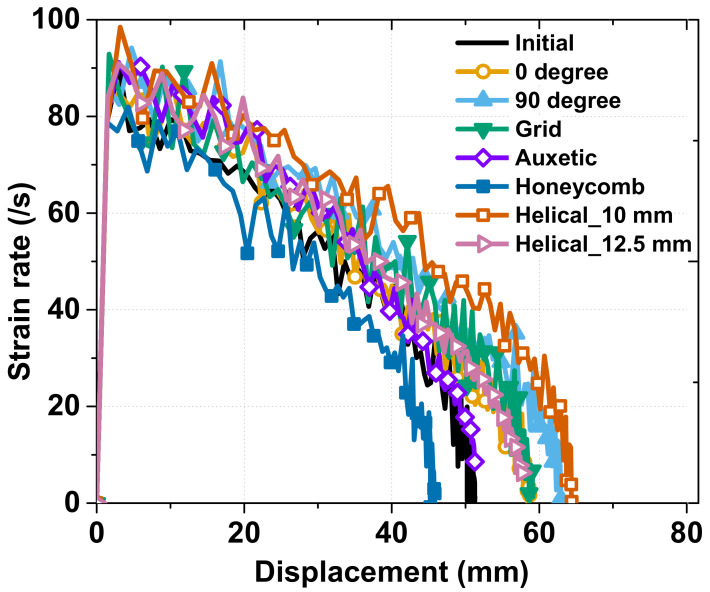
Strain rate according to displacement during dynamic crash test.

In this section, we discuss the strain rate sensitivity under dynamic conditions for the same stroke of 40 mm. In the strain rate comparison of the various patterns shown in Figure 9, the helical and 90° patterning specimens exhibited the best results, which is in good agreement with the total absorbed energy and axial crash force, as mentioned above.

The largest strain rate reduction was observed for the honeycomb patterning specimens, while the helical and 90° patterning specimens exhibited the smallest reduction because the honeycomb and helical patterning specimens, respectively, had the smallest and largest displacements with the same axial crash–force peak value, as shown in Figure 9. The reason why the honeycomb had the same axial crash force peak value but a considerably smaller displacement value than the helical patterning specimens is discussed below. At the third peak force, the honeycomb patterning specimen folded into a diamond shape, and it expanded along the transverse direction with respect to the loading direction [25], which caused the tube displacement to stop under the crashing force. In contrast, the helical patterning specimens did not attain such a special structure, and they thus gradually absorbed all the crash energy, which resulted in the greatest displacement. In the case of the auxetic structure, although it had a similar strain rate according to the displacement value as the original tube, the first and second peak values of the axial crash force were as high as those of the honeycomb and helical patterning specimens. This phenomenon was due to the negative Poisson’s ratio of the auxetic structure, owing to which the structure tends to shrink along the lateral direction, as illustrated by Lee et al. [25]. Therefore, the auxetic structure also balances three values: the highest peak axial crash forces, a considerably high amount of absorbed energy, and a moderate displacement. The strain rate according to the displacement of the other patterns showed good agreement with the axial crash test results shown in Figure 8; the greater the value of the total displacement, the smaller the strain rate reduction was.

Based on Figure 8 and Figure 9, we can determine the appropriate patterning for a suitable application. If we need to maximize the absorbed energy, the 0°, 90°, and helical patterns are promising candidates; conversely, the honeycomb pattern is the most suitable for an application wherein the displacement should be limited, for example, the bumper of a small and compact vehicle. The auxetic pattern is selected when a balance is required between the absorbed energy and deformed area.

### 3.3. Optimum Design in Dynamic Crash Test

**Figure 10:**
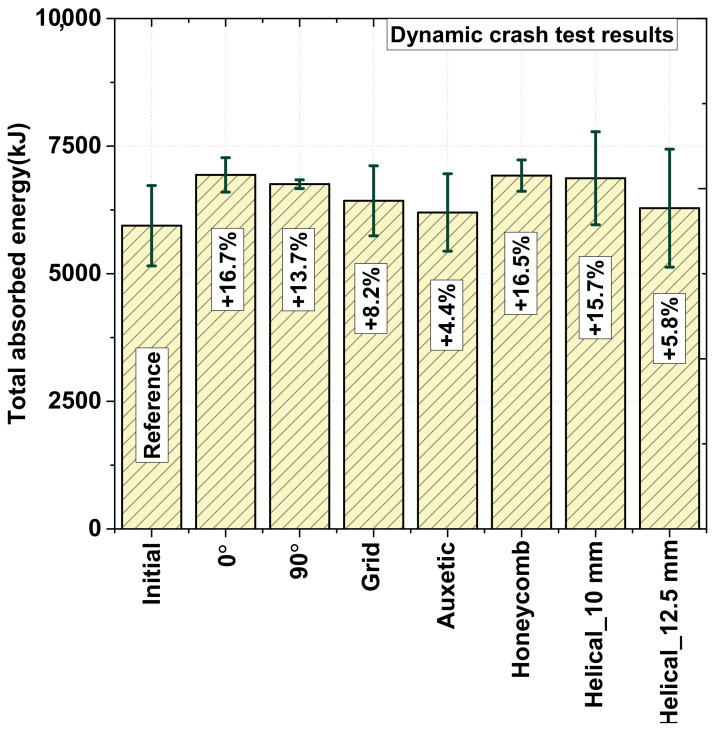
Energy absorption with dynamic crashing conditions at 40 mm.

**Figure 11:**
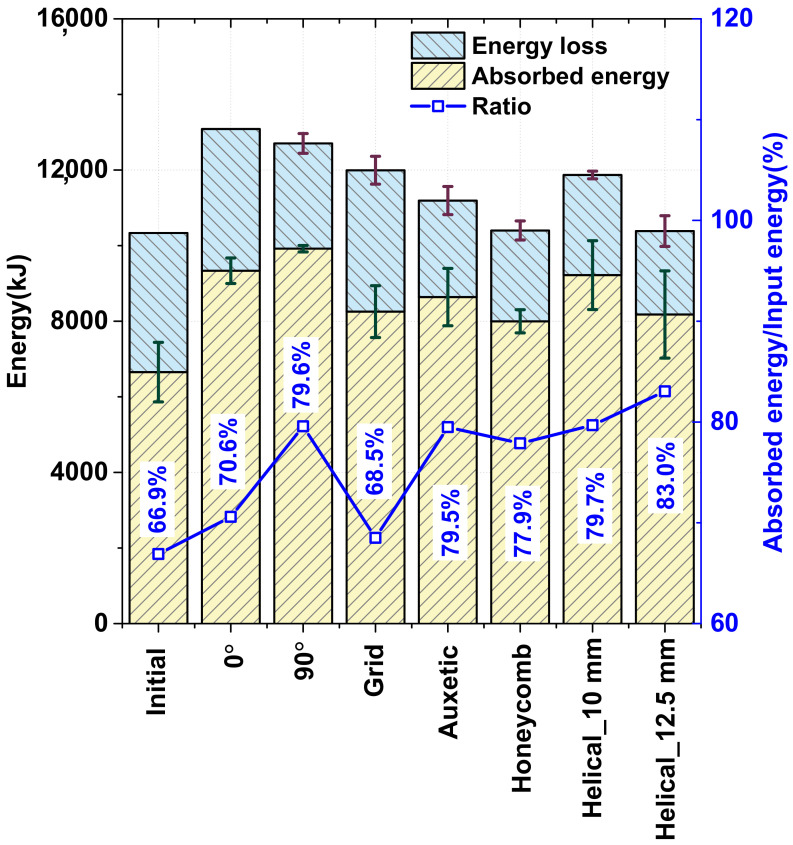
Performance comparison betwen dynamic crash tests according to laser patterning shape.

The improvement in the total absorbed energy for different patterns is presented in Figure 10. Owing to the increase in the oil temperature, the speed of the hydraulic tester could not be precisely controlled and fluctuated during the testing, which caused an inaccurate performance evaluation. For obtaining an equal comparison, the method of comparing the ratio of the absorbed energy per input energy, called the “absorbed energy ratio”, is presented in Figure 11. According to Figure 11, the optimal (helical patterning specimen with a 12.5-mm pitch) specimen provided the best result with an absorbed energy ratio of up to 80.3%, which is considerably higher than that of the other patterning types. The auxetic, 90°, and helical patterning specimens were among the second group, which had an approximate absorbed energy ratio ranging from 79.5% to 79.7%. By performing a comparison in this manner, the result was found to exhibit the same trend as that of the static loading test result, wherein the optimal pattern exhibited the best crashing performance [3].

As described in a previous study [3], the 90° patterning specimen exhibited severe cracks along the laser patterning area after the first peak, resulting in low absorption of the deformation energy; however, in the dynamic crash test, this phenomenon was not observed. Owing to the increased hardening during the dynamic impact, the strain rate increased, as shown in Figure 9, and it could be predicted that the fracture of the 90° patterning specimen would not occur because it could withstand a greater force in the dynamic crash test.

**Figure 12:**
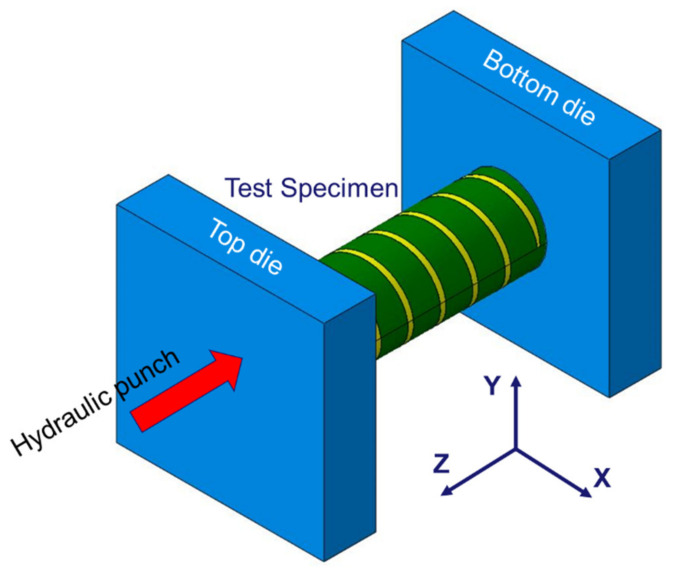
Model setup in FEM analysis.

**Figure 13:**
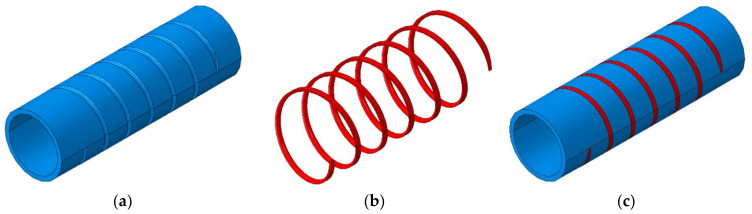
Helical specimen modelling technique in ABAQUS: (**a**) initial 22 MnB5 steel tube; (**b**) Martensite helical pattern; (**c**) Tube with Helical pattern.

To validate the design and experimental results of the optimal specimens and other patterning types, an FEM analysis using ABAQUS/Explicit was performed, and the detailed model is illustrated in Figure 12 using Catia V5 to create complex laser patterning shapes. In the case of the helical patterning specimens, the tube was divided into two parts with different materials, as shown in Figure 13, those being the tube with a base material (22 MnB5 steel) and the patterning part with martensite steel after laser local hardening. The two parts were assembled using the “tie constraint”. The bottom die was fixed while the top die and punch were moving at the initial velocity that varies between 7 m/s and 9 m/s according to each test case listed in Table 2.

In the case of the meshing technique, a hexahedral element type of an approximate size of 0.5 mm is applied to all the specimens, except the helical and optimal specimens, and a total of approximately 245,000 elements of type C3D8R were used. Owing to the complex geometry of the helical and optimal specimens, to which the hexahedral element could not be applied, the tetrahedral element was applied with an element size of 0.5 mm, and a total of 408,000 elements of the C3D10M type were used. The hexahedral element of size 2 mm was also used for the bottom and top dies, and the total number of elements of each part was 5100 and 4800, respectively.

**Figure 14:**
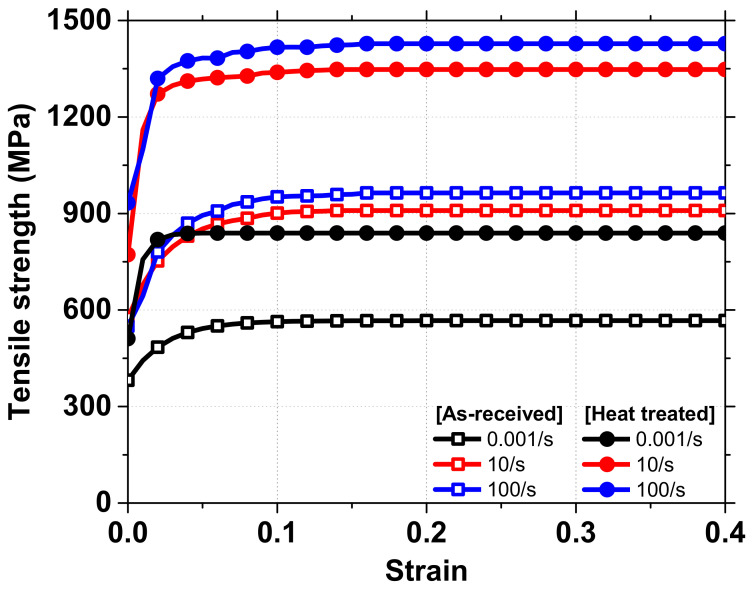
Tensile strength at different strain rate of As-received Material and Material after Heat treated.

**Figure 15:**
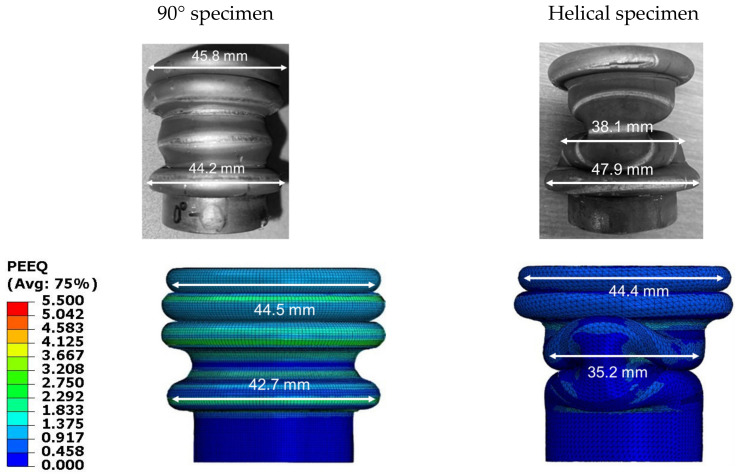
Deformation mode in FEM analysis.

A stress–strain curve with respect to the 0.001, 10, and 100/s of strain rate was obtained through the tensile test with as-received and heat treatment specimens, in order to confirm the strain rate sensitivity shown in the dynamic crash test, and it is shown in Figure 14. The deformation mode in the FEM analysis is presented in Figure 15. Overall, the deformation mode result in FEM result shows good correlation with the experiment. Regarding the 90° specimen, all of four folds undergoing FEM analysis are concertina mode while the experiment only has three folds. The last fold of experiment specimen is in the middle of the tube, if we have more, kinematic energy, it will transform into the concertina mode. In the FEM analysis, due to no friction, all the kinetic energy is converted into the deformation energy, thus making more folds in the concertina mode. The FEM analysis and experiment show the same results concerning the helical patterns, which are two folds with concertina mode and a one-fold-having diamond mode. The difference in position of second peak with concertina mode between FEM and experiment occurred because FEM analysis did not include damping value; the material of tube and material of heat-treated area were not uniform perfectly, as in the simulation environment. 

**Figure 16:**
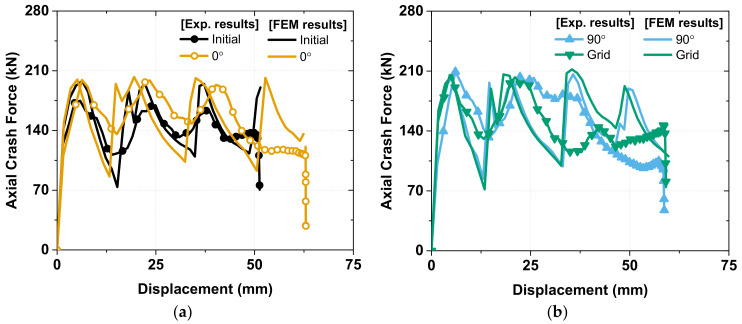

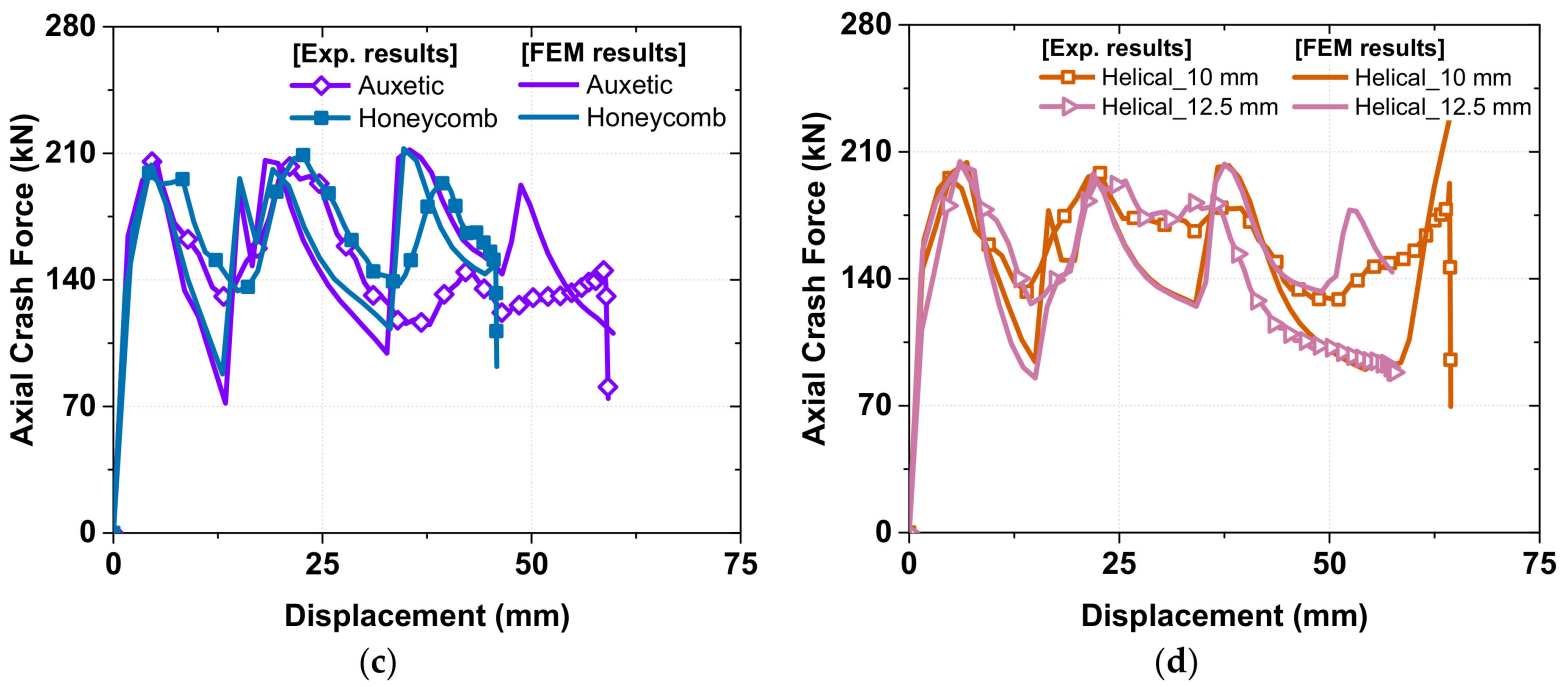
Comparing Axial crash force between FEM analysis and Experiment: (**a**) Initial and 0°; (**b**) 90° and Grid; (**c**) Auxetic and Honeycomb; (**d**) helical with pitch of 10 mm and helical with pitch of 12.5 mm.

**Figure 17:**
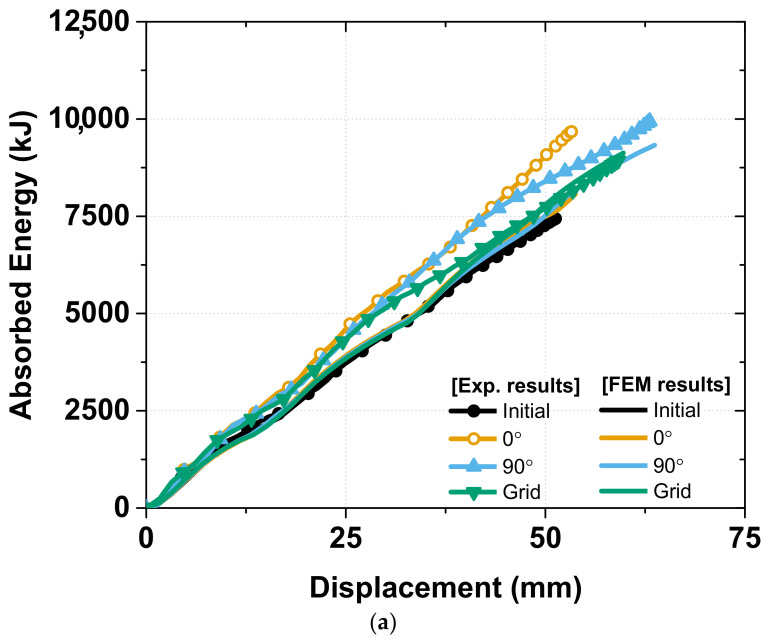

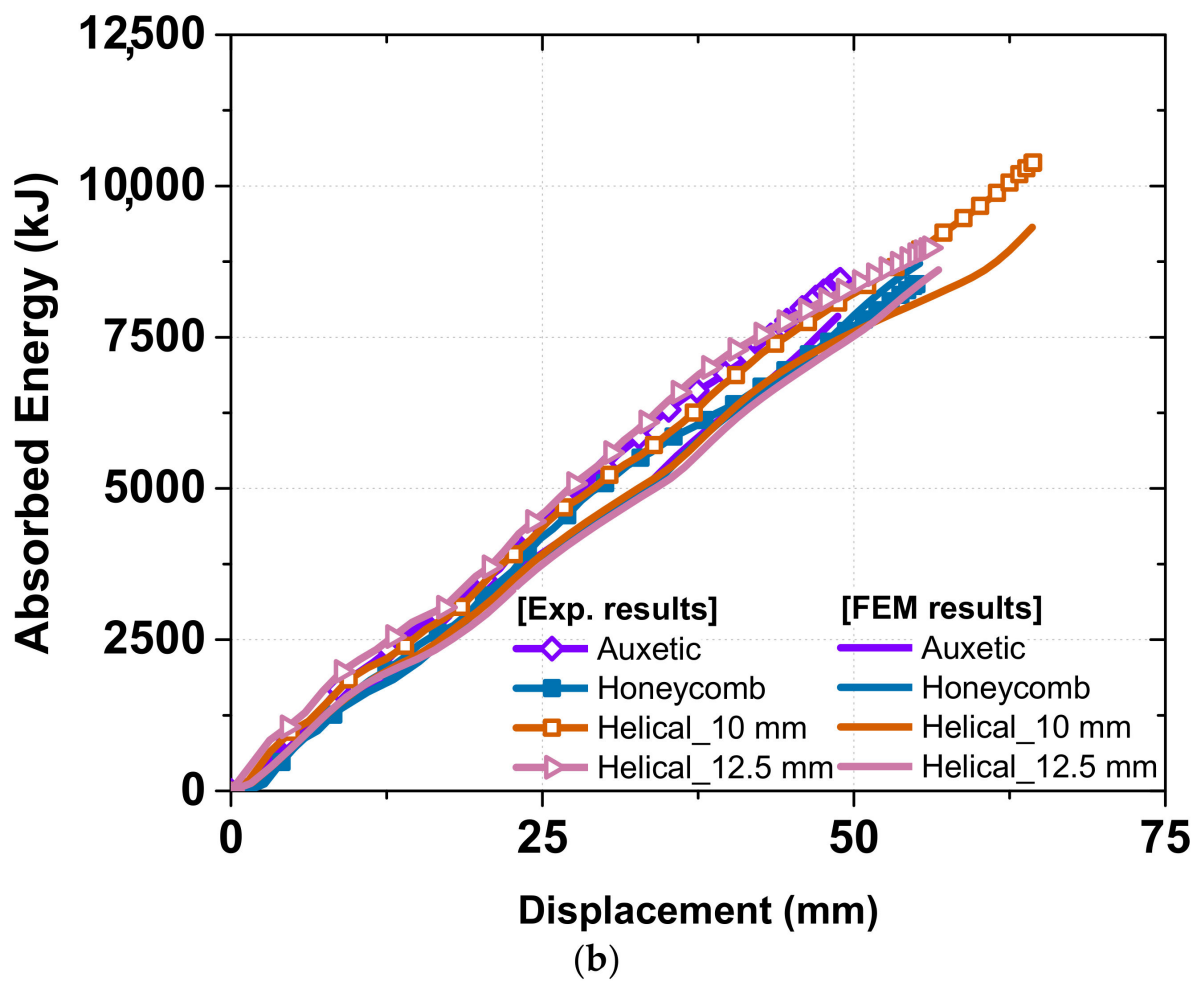
Comparing Total absorbed energy between FEM analysis and Experiment: (**a**) Group 1: initial, 0°, 90° and grid; (**b**) Group 2: auxetic, honeycomb, helical with pitch of 10 mm and helical with pitch of 12.5 mm.

**Figure 18:**
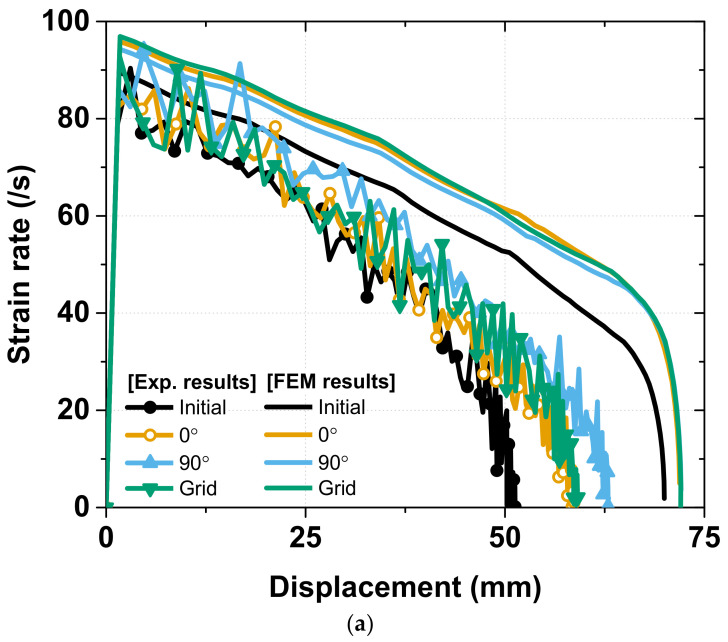

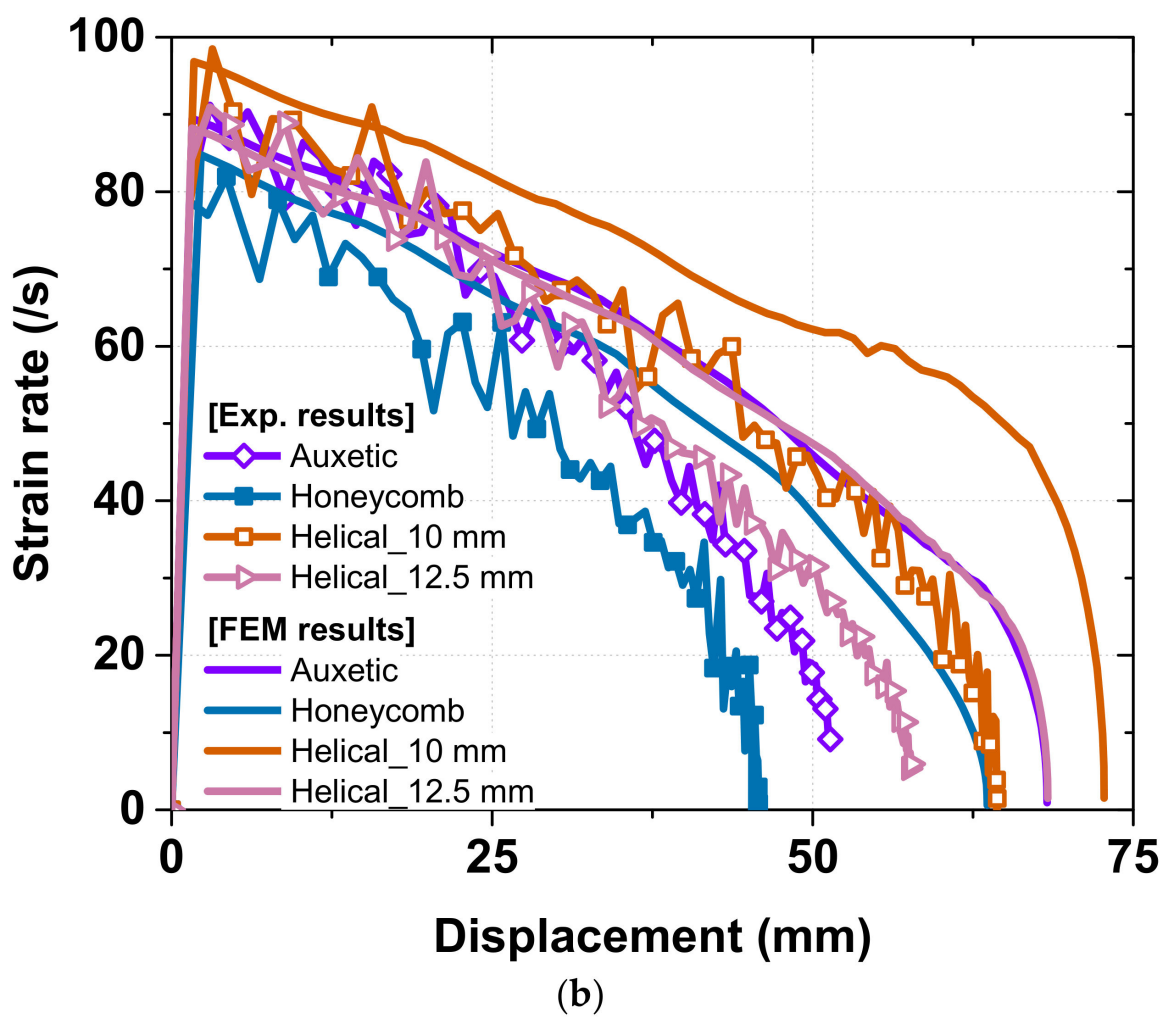
Comparing Strain rate between FEM analysis and Experiment: (**a**) Group 1: initial, 0°, 90° and grid; (**b**) Group 2: auxetic, honeycomb, helical with pitch of 10 mm and helical with pitch of 12.5 mm.

In general, the FEM analysis results exhibited a good correlation with the experimental results for the axial crash force (in Figure 16), absorbed energy (in Figure 17), and strain rate sensitivity (in Figure 18). In the experiment there are tolerances that occur during the manufacturing process of specimens and die, but FEM analysis does not consider tolerances in modeling. As shown in Figure 16, this difference in tolerance makes the FEM analysis results sharper than the experimental results, the details of which are discussed below.

In the case of the axial crash force, the FEM analysis presented the same peak and minimum crash force with values of 210 kN and 100 kN in the first and second fold for all the specimens. In the case of the third fold, the FEM results exhibited a higher value than the experimental result because, in the experiment, the hydraulic tester had friction between several of its moving parts, which caused energy dissipation and crashing speed fluctuation.

With respect to the absorbed energy, the FEM analysis result exhibited a good correlation in both the groups with the experiment with a peak value of approximately 10,500 KJ, and the helical patterning specimen exhibited the best performance in absorbing the crash energy.

In terms of the strain rate sensitivity, there was a good tendency between the FEM analysis and the experiment for all the groups. The peak strain rate of approximately 100/s was observed for the helical patterning specimen, while the other specimen only achieved a peak strain rate of 85/s, and there were no ripples in the FEM analysis chart owing to the consideration of an ideal crash condition with a constant impact force. The total displacement of the specimen in the FEM analysis was also higher than that in the experiment by approximately 10 mm because, with the same initial kinetic energy in the simulation environment with no energy dissipation, the impact tester could cause more deformation.

### 3.4. Discussion

The reason why the 90°, optimal, helical, and auxetic patterning specimens were the group that exhibited the best performance (Group 1) in energy absorbance is discussed in this section. The first reason is the deformation mode shape and the number of folds: all the patterns of Group 1 had three folds, while some of the others in Group 2 only had two folds, e.g., honeycomb and grid patterning specimens.

Although the honeycomb and grid patterning specimens only had two folds, the honeycomb specimen had a considerably higher absorbed energy ratio than the grid specimen owing to its special structure. The 90° patterning specimen exhibited a significant improvement in the dynamic test as compared with the static test mentioned in the study by Kim et al. [3] because a crack did not occur under the dynamic effect.

The second reason is related to the uniformity in the lateral confining pressure distribution, which was discussed by Kim et al. [3]. Although the auxetic specimen only had two folds in the concertina mode, its performance was notable, similar to those of the 90° and helical patterning specimens, and only lower than that of the optimal specimen because of the advantage of the negative-Poisson’s-ratio structure in absorbing force [25].

In the previous research [3], we confirmed that the energy absorption of the helical pattern was improved by 22.3% compared to the as-received specimen, and it was verified when using the optimally designed specimen with a pitch size of 12.5 mm, the energy absorption was improved by 32.2% through experiments and numerical simulation. This trend also occurred in dynamic crash test, and it was confirmed in this paper that the ratio of absorbed energy to input energy was improved by 13.1% when using the optimally designed specimen with a pitch size of 12.5 mm compared to the as-received specimen. The optimal specimens exhibited the best performance because of the matching of the patterning position with the folded area; in this case, the pitch of the helical pattern was equal to the line length of a single fold along the neutral surface after the initial tube collapsed. Thus, to achieve the highest absorption energy, there was a combination of the number of folds, deformation mode, patterning structure, and the position of the heat-treated area that could be implemented.

## 4. Conclusions

In this study, the effect of laser patterning on local strengthening induced by martensitic transformation during an axial crash test, in terms of the deformation mode and energy absorption, was validated via experiments and finite element analyses under dynamic conditions using a 22 MnB5 steel tube. The laser patterning only has a slight effect on the deformation mode at a high strain rate because all the specimens deformed in the concertina mode during the first folding. The case where the pitch size of the helical pattern was optimized showed the largest energy absorption rate of 83%, and the initial helical pattern and auxetic pattern showed very good energy absorption rates of 79.7 and 79.5%. Although the 90° patterned specimen exhibited a poor performance in energy absorbance in the static crash test, it exhibited a significant improvement under dynamic conditions. This is because cracks did not occur. In contrast to the static crash test condition, the deformation mode was not a dominant factor that affected the energy absorbance of the patterned specimen. To maximize the energy absorbance, there was a combination of many factors such as the number of folds, position of the heat-treated area, and patterning structure that could be implemented. If the impacting velocity fluctuates because of the tester, the use of the ratio of the absorbed energy and input energy is recommended to appropriately assess the energy absorbance performance.

Finally, the helical patterning specimen exhibited the best performance in both the static and dynamic tests. The optimal design in this case was the helical pattern with a pitch of 12.5 mm, which is equal to the folding diameter. By measuring the line length of a single fold along the neutral surface when the as-received tube collapsed after the crash test, we could define the suitable pitch of the helical pattern to fit the tube geometry.

## Figures and Tables

**Table 1:** Deformation mode according to folding in each patterning shape.

	Initial	0°	90°	Grid	Auxetic	Honeycomb	Helical_10 mm	Helical_12.5 mm
1st fold	C	C	C	C	C	C	C	C
2nd fold	C	C	C	D	C	D	C	C
3rd fold	C	C	D	None	D	None	D	C

**Table 2:** Speed of Hydraulic punch in each test case.

Specimen	Speed (m/s)
Initial	8.21
0°	8.79
90°	8.65
Grid	8.89
Auxetic	8.21
Honeycomb	7.8
Helical_10 mm	8.89
Helical_12.5 mm	8.1

## Data Availability

Not applicable.
